# Diagnostic challenge of dyspnea in the context of the COVID-19 pandemic wave: a case report

**DOI:** 10.1186/s43162-023-00192-6

**Published:** 2023-01-31

**Authors:** Zamelina Angela Razafindrasoa, Sonia Marcelle Razafimpihanina, Marie Odette Rasoafaranirina, Fidy Arnauld Martin, Finaritra Princy Parfait Andriamahenina, Diamondra Ombanjanahary Andriarimanga, Jocelyn Robert Rakotomizao, Harison Michel Tiaray, Joëlson Lovaniaina Rakotoson, Rondro Nirina Raharimanana

**Affiliations:** 1 Centre Hospitalier Universitaire Fenoarivo, Antananarivo, Madagascar; 2 Centre Hospitalier Universitaire Tambohobe, Fianarantsoa, Madagascar; 3 Centre Hospitalier Universitaire Joseph Raseta Befelatanana, Antananarivo, Madagascar

**Keywords:** Case report, COVID-19, HIV, Infection, Madagascar

## Abstract

**Background:**

Since its discovery, COVID-19 has often been the first diagnosis of dyspnea and asthenia, especially during the pandemic waves. However, it is not always COVID-19. We report a particular case of a late-diagnosed HIV-positive patient in Madagascar.

**Case presentation:**

A 21-year-old male patient was admitted to a hospital center in Antananarivo for dyspnea and poor general condition. Physical examination revealed hypoxemia of 85% on room air. His chest X-ray showed bilateral reticular-micronodular opacities. He was suspected and treated for COVID-19. On the 15th day of hospitalization, HIV-1 infection complicated by probable pneumocystis was diagnosed. On the other hand, a multimetastatic testicular cancer was also suspected. The patient died after a few hours of hospitalization in the intensive care unit.

**Conclusion:**

This was a case of an HIV-positive patient belatedly diagnosed in the complications stage during the COVID-19 pandemic wave. The investigation of the differential diagnoses remains crucial to avoid serial misdiagnosis and to adjust therapeutic management.

## Background

Coronavirus disease 2019 (COVID-19) due to severe acute respiratory syndrome coronavirus 2 (SARS-CoV-2) has remained at the forefront of public health and medical practice since its discovery in December 2019. Currently, this disease is often at the top of the differential diagnoses in front of dyspnea and asthenia [1]. Thus, other diagnoses are easily missed [2, 3]. We report a special case of a late-diagnosed human immunodeficiency virus (HIV)-positive patient presenting with dyspnea and lung lesions during a wave of COVID-19 in Madagascar.

## Case presentation

The patient was a Malagasy 21-year-old male, a street vendor. He consulted his treating physician for a flu-like syndrome for one month. Despite the treatments he received, he developed an exertional dyspnea evolving in the context of asthenia and emaciation. Hence his reference to the pulmonology department of Centre Hospitalier Universitaire Fenoarivo, Antananarivo. He had no specific medical history until 4 months before hospitalization. Indeed, he had a history of anal condyloma 4 months prior to hospitalization, which was operated on a doctor’s office 2 months later, without exploration. In his sexual history, we noted that he was homosexual.

On admission, the pulse oxygen saturation (SpO_2_) was 85% on room air but his hemodynamic status was stable. His body mass index was 15.6 kg/m^2^. Respiratory examination showed signs of moderate respiratory distress and audible crepitations upon basal pulmonary auscultation. Heart sounds were normal. The abdomen was soft but tender on palpation of the right hypochondrium. Examination of the anal sphincter revealed perianal sores. Examination of the external genitalia found the loss of scrotal rugae, which was not painful to palpation.

The blood tests showed a biological inflammatory syndrome. Table 1 summarizes the results of the initial biological tests performed.

**Table 1:** Biological test results

Biological tests	Results	
Blood cell count		
Leukocytes	7140/mm^3^	Normal
Hemoglobin	11.4 g/dl	Normal
Platelets	155,000/mm^3^	Normal
C-reactive protein	65 mg/l	Elevated
D-Dimers	1.9 mg/l	Elevated
Glycemia	3.5 mmol/l	Normal
Creatininemia	92 µmol/l	Normal
Transaminasemia		
SGPT (serum glutamic pyruvic transaminase)	16 UI	Normal
SGOT (serum glutamic-oxaloacetic transaminase)	18 UI	Normal
Total protein level	60 mg/l	Normal
Sputum examination for acid-fast bacilli	Negative	
SARS-CoV-2 antigen test (SD Biosensor® Republic Korea)	Negative	

**Fig. 1:**
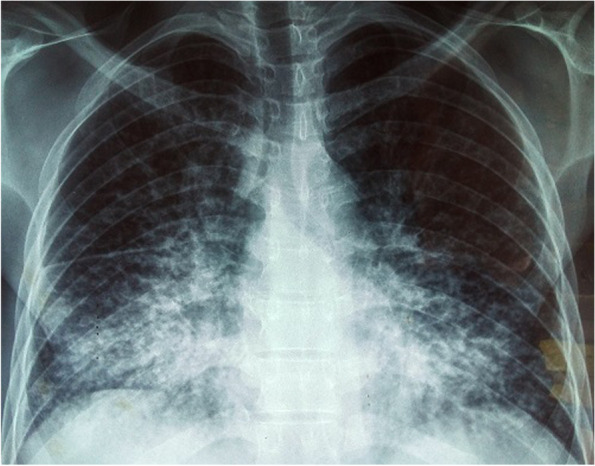
Front view chest X-ray showing reticular-micronodular opacities in the hilar and basal pulmonary regions

Chest X-ray showed reticular-micronodular opacities in the hilar and basal lung regions, bilaterally (Fig. 1). Chest CT scan and bronchial fibroscopy were not available.

In view of the oxygen desaturation and the unexplained interstitial syndrome during the COVID-19 wave, he was treated as a case of severe COVID-19 by azithromycin 500 mg on day 1 and 250 mg/day for the next 4 days. Ceftriaxone 2 g/day, dexamethasone 8 mg/day, and enoxaparine 0.4 mg/day for 14 days.

After 3 days of treatment, the evolution seemed favorable with a SpO2 of 91% on 1 l/min oxygen. However, by day 5 he had become dyspnoeic again, requiring an increase in oxygen therapy from 2 to 4 l/min for 10 days to reach a SpO2 of 95%. No workup could be done because of the lack of financial means from the patient. The evolution was stationary until the 15th day when the patient became agitated, tachypneic, and complained of unspecified chest pain, and intense abdominal-anal and scrotal pain. SpO_2_ was 91% on 8 l/min oxygen. The vital parameters were normal with a blood pressure of 120/80 mmHg, heart rate of 80 beats per minute, and temperature of 36.8 °C. The clinical probability score for pulmonary embolism was low. The second-line biological tests revealed an HIV infection (Table 2). The CD4 count and viral load were being measured. The HBs antigen test was negative. Testicular ultrasound showed a large inflammatory infiltration of the intrascrotal fat bilaterally, associated with an intravaginal effusion slide, without evidence of torsion or testicular nodule. Abdominal ultrasonography revealed a multinodular hepatomegaly of 178 mm, the largest of which measured 6 mm in diameter, associated with latero-aortic lymphadenopathy. Thus, testicular cancer with interstitial lung, liver, and lymph node metastasis was suspected. However, tumor marker assays could not be performed. The patient's condition did not allow for immediate diagnostic and therapeutic testicular surgery.

**Table 2:** HIV testing results

Rapid HIV testing	Reactive
HIV serology (ELISA test confirmed by Western blot)	
HIV-1	Positive
HIV-2	Negative

In view of the febrile dyspnea in an immunocompromised patient, antibiotic therapy for Pneumocystis jirovecii was started, with the only medicine that exists in the country, sulfamethoxazole-trimethoprim 800/160 mg, 2 tablets 3 times a day.

After a few hours of hospitalization in the intensive care unit, the patient died of disseminated intravascular coagulopathy.

## Discussion

In total, our case was about an HIV/AIDS (acquired immunodeficiency syndrome) disease with probable opportunistic pneumocystis infection and multimetastatic testicular cancer. The patient’s case ended in death after four and a half months of misdiagnosis in the context of the COVID-19 pandemic wave.

Our patient was suspected and treated for COVID-19 even though the antigenic test was negative. Indeed, antigen tests for SARS*-*CoV*-*2 are generally less sensitive than real-time reverse transcription polymerase chain reaction (RT-PCR). In cases of a lack of RT-PCR, particularly during pandemic waves, the national protocol for the management of COVID-19 recommends the treatment of any clinically suspected case.

Regarding the diagnosis of HIV infection, the delay is probably linked to the impact of COVID-19. Indeed, the two diseases have similar clinical symptoms. Nomah DK and allies discussed that the increased channeling of care to COVID-19 decreased access to early HIV diagnosis [4]. However, the discovery of a condyloma should prompt a search for a sexually transmitted infection.

After HIV diagnosis, pulmonary pneumocystis was suspected on clinical and radiological grounds. Pneumocystis jirovecii pneumonia is a common and potentially serious opportunistic infection in HIV-positive patients with a CD4 count < 200/mm^3^ [5]. The clinical presentation generally includes fever, dyspnea with hypoxemia, and nonproductive cough. Chest X-ray or CT scan are important for establishing the diagnosis, as biological confirmation is difficult to obtain. Lesions may present as a micronodule or an interstitial ground-glass syndrome [6].

Although we have no biological or histological evidence, multimetastatic testicular cancer has been suspected on epidemiological-clinical-radiological grounds. Indeed, testicular cancer is the leading cancer in young men aged 20–35 years [7]. It metastasizes naturally through the lymphatic and hematogenous routes, giving rise to rapid lung, liver, and lymph node metastases. There are several risk factors, including cryptorchidism and HIV infection [8]. The correlation between HIV-induced immunosuppression and increased cancer risk has long been known [9]. Signs of cancer include inflammation of the testicle, a feeling of heaviness in the scrotum, and fluid accumulation in the scrotum. Scrotal ultrasound is important. Chest radiography looks for pulmonary metastatic lesions in the form of bilateral basal micronodular opacities. Histological examination provides diagnostic certainty [10].

Madagascar is a very low-income country. In terms of health care, the performance of complementary examinations is often limited by the unavailability of tests and/or the financial situation of patients. The autopsy of the patient would have offered a precise, complete diagnosis post-mortem. Unfortunately, it was not performed, and it is not yet common in routine medical practice in our country.

## Conclusion

Hypoxemia and lung lesions during the COVID-19 pandemic wave do not always mean SARS-CoV-2 infection. HIV infection is one of the differential diagnoses that medical providers should screen in any at-risk patient. HIV-related opportunistic infections and cancers are serious. Unfortunately, the investigations were limited in our case. However, physicians should not rush for therapeutic management before a definite diagnosis. In Madagascar, medical, social, and economic efforts must be made to improve patient outcomes.

## Data Availability

The data that support the findings of this study are not publicly available due to privacy concerns. The data are available from the corresponding author upon reasonable request.

## Abbreviations

AIDS: Acquired immunodeficiency syndrome
COVID-19: Coronavirus disease 2019
HIV: Human immunodeficiency virus
SARS-CoV-2: Severe acute respiratory syndrome coronavirus 2
SGPT: Serum glutamic pyruvic transaminase
SGOT: Serum glutamic-oxaloacetic transaminase
SpO_2_: Pulse oxygen saturation
